# Relapse Rates and Disease-Specific Mortality Following Procedures for Fertility Preservation at Time of Breast Cancer Diagnosis

**DOI:** 10.1001/jamaoncol.2022.3677

**Published:** 2022-08-25

**Authors:** Anna Marklund, Tobias Lekberg, Elham Hedayati, Annelie Liljegren, Jonas Bergh, Frida E. Lundberg, Kenny A. Rodriguez-Wallberg

**Affiliations:** 1 Department of Oncology-Pathology, BioClinicum, Karolinska Institutet, Solna, Sweden; 2 Division of Gynecology and Reproduction, Department of Reproductive Medicine, Karolinska University Hospital, Stockholm, Sweden; 3 Breast Cancer Theme Center, Karolinska University Hospital and Karolinska Comprehensive Cancer Centre, Stockholm, Sweden; 4 Department of Internal Medicine, Southern Hospital, Stockholm, Sweden; 5 Department of Medical Epidemiology and Biostatistics, Karolinska Institutet, Sweden; 6 Laboratory of Translational Fertility Preservation, BioClinicum, Stockholm, Sweden

## Abstract

**Importance:**

Breast cancer (BC) is the most common indication for fertility preservation (FP) in women of reproductive age. Procedures for FP often include hormonal stimulation, but current data are scarce regarding whether using hormonal stimulation for FP is associated with any deterioration in BC prognosis.

**Objective:**

To investigate the risk of disease-specific mortality and relapse in women who underwent FP with or without hormonal stimulation compared with women who did not at time of BC diagnosis.

**Design, Setting, and Participants:**

This Swedish nationwide prospective cohort study was conducted to assess the safety of hormonal and nonhormonal FP procedures indicated by BC in Sweden from January 1, 1994, through June 30, 2017. Women were identified from any of the regional FP programs located at Swedish university hospitals. A total of 425 women were found to have undergone FP, and 850 population comparators who had not undergone FP were sampled from regional BC registers and matched on age, calendar period of diagnosis, and region. Relapse-free survival was assessed in a subcohort of 241 women who underwent FP and 482 women who had not, with complete data. Nationwide demographic and health care registers provided data on outcome, disease- and treatment-related variables, and socioeconomic characteristics. Data analyses were performed between November 2021 and March 2022 and completed in June 2022.

**Main Outcomes and Measures:**

Relapse and disease-specific mortality after a diagnosis of BC.

**Results:**

The final study population included 1275 women (mean [SD] age, 32.9 [3.8] years) at the time of BC diagnosis. After stratification by the matching variables age, calendar period, and region, and adjustment for country of birth, education, parity at diagnosis, tumor size, number of lymph node metastases, and estrogen receptor status, disease-specific mortality was similar in women who underwent hormonal FP (adjusted hazard ratio [aHR], 0.59; 95% CI, 0.32-1.09), women who underwent nonhormonal FP (aHR, 0.51; 95% CI, 0.20-1.29), and women who were not exposed to FP (reference). In a subcohort with detailed data on relapse, adjusted rate of disease-specific mortality and relapse were also similar among the groups who underwent hormonal FP (aHR, 0.81; 95% CI, 0.49-1.37), underwent nonhormonal FP (aHR, 0.75; 95% CI, 0.35-1.62), and were not exposed to FP (reference).

**Conclusions and Relevance:**

In this cohort study, FP with or without hormonal stimulation was not associated with any increased risk of relapse or disease-specific mortality in women with BC. Results of this study provide much needed additional evidence on the safety of FP procedures in women with BC and may influence current health care practice to the benefit of young women with BC who wish to preserve their fertility.

## Key Points

**Question:**

Is fertility preservation at time of breast cancer diagnosis associated with any increased risk of disease-specific relapse or mortality?

**Findings:**

In this population-based Swedish nationwide cohort study that included 1275 women with breast cancer, fertility preservation at time of breast cancer diagnosis was not statistically significantly associated with any increased risk of disease-specific mortality or relapse.

**Meaning:**

Findings of this study support the safety of fertility preservation in women with breast cancer, which is highly relevant for reproductive counseling of women with breast cancer diagnosed at a young age.

## Teaser

This cohort study investigates the risk of disease-specific mortality and relapse in women who underwent fertility preservation with or without hormonal stimulation compared with women who did not at time of breast cancer diagnosis.

## Introduction

Breast cancer (BC) is the most common cancer in young women,^1^ and it usually presents with more aggressive biological features and more advanced stage at diagnosis than in older women.^2,3,4,5^ Because these factors may lead to a potentially less favorable prognosis, young women generally receive more intensive treatment.^6,7,8^ Current protocols of BC chemotherapy have improved survival rates, but they may also lead to infertility.^9,10^ Additionally, women with hormonally sensitive cancers are advised to delay pregnancy until completion of 5 to 10 years of adjuvant endocrine therapy,^11,12^ which often results in the natural age-related fertility decline.^13^

Infertility due to cancer treatment is an important issue for patients diagnosed with cancer at reproductive age.^14,15^ There are clear recommendations about timely information to patients on potential infertility risk inherent to cancer treatment and about the possibility to undergo fertility preservation (FP).^16^ Currently, established methods for adult female patients include cryopreservation of embryos, oocytes, and ovarian tissue.^17^ Cryopreservation of embryos and/or oocytes can be offered when the patient’s condition allows them to undergo a controlled ovarian stimulation (COS) treatment, requiring about 2 weeks to complete.^17^

Controlled ovarian stimulation treatments result in supraphysiologic levels of circulating estradiol,^18^ which could theoretically stimulate tumor growth and increase the risk of dissemination in women with hormone-sensitive BC.^19,20^ Although studies so far have not found higher risks of BC relapse or mortality in women undergoing COS,^21,22,23^ relatively short follow-up times and the small number of included patients render caution in the interpretation of the results. Potentially safer stimulation protocols with coadministration of tamoxifen and letrozole to suppress estradiol levels during COS have been developed,^24,25^ with the latter drug reported to have higher efficacy and is, therefore, currently implemented at many centers.^26^ So far there is no evidence that these protocols provide benefits in terms of safety when compared with standard COS.^27^ The largest published study on safety of COS with letrozole in women with BC compared that protocol with no FP procedures, concluding that the use of letrozole-based protocols was not associated with any increased risk of deteriorated survival.^28^

The need for studies investigating BC disease-specific outcomes following FP in larger cohorts with long-term follow-up has been highlighted in previous reports.^21,23^ The aim of this cohort study was to investigate the long-term safety of FP with and without hormonal stimulation in a large Swedish nationwide cohort of young women with BC by comparing disease-specific mortality and relapse rates in women who had undergone FP at the time of their BC diagnosis with women who had not.

## Methods

### Data Sources and Study Population

This is a population-based nationwide cohort study of women diagnosed with BC at reproductive age (herein defined as ages 18-44 years) in Sweden between 1994 and 2017. The cohort and the methodology have been described previously.^29^ Briefly, women with BC who had undergone FP between January 1, 1994, and June 30, 2017, at any of the regional FP programs located at Swedish university hospitals were identified. Data on FP procedures were extracted from the electronic medical records of each hospital. This study was approved by the regional ethics committee in Stockholm, Sweden (Dnr 2011/1758-31/2; amendments Dnr 2014/470-32, Dnr 2014/1360-32, Dnr 2014/1825-32, Dnr 2018/275-32, and Dnr 2018/1453-32). Patient consent was obtained orally at time of FP until 2008, when it was obtained thereafter in writing. This study followed the Strengthening the Reporting of Observational Studies in Epidemiology (STROBE) reporting guidelines.

Three Swedish quality registers for BC (eTable 1 in the Supplement) were used to create a matched cohort. For each woman exposed to FP, 2 population comparators with BC but unexposed to FP were randomly selected by matching on health care region, age at diagnosis (within a 5-year range), and calendar period at diagnosis (within a 3-year range). For women diagnosed between 2008 and 2017, data were obtained from the Swedish National Quality Register for BC, as this register was initiated in 2008.^30^ Data on women diagnosed in 2007 or earlier were obtained from the regional BC registers for Stockholm-Gotland and West regions. Only 3 women outside of these regions were exposed to FP before 2008, and these women were excluded because we were unable to sample comparators to them.

**Figure 1.**
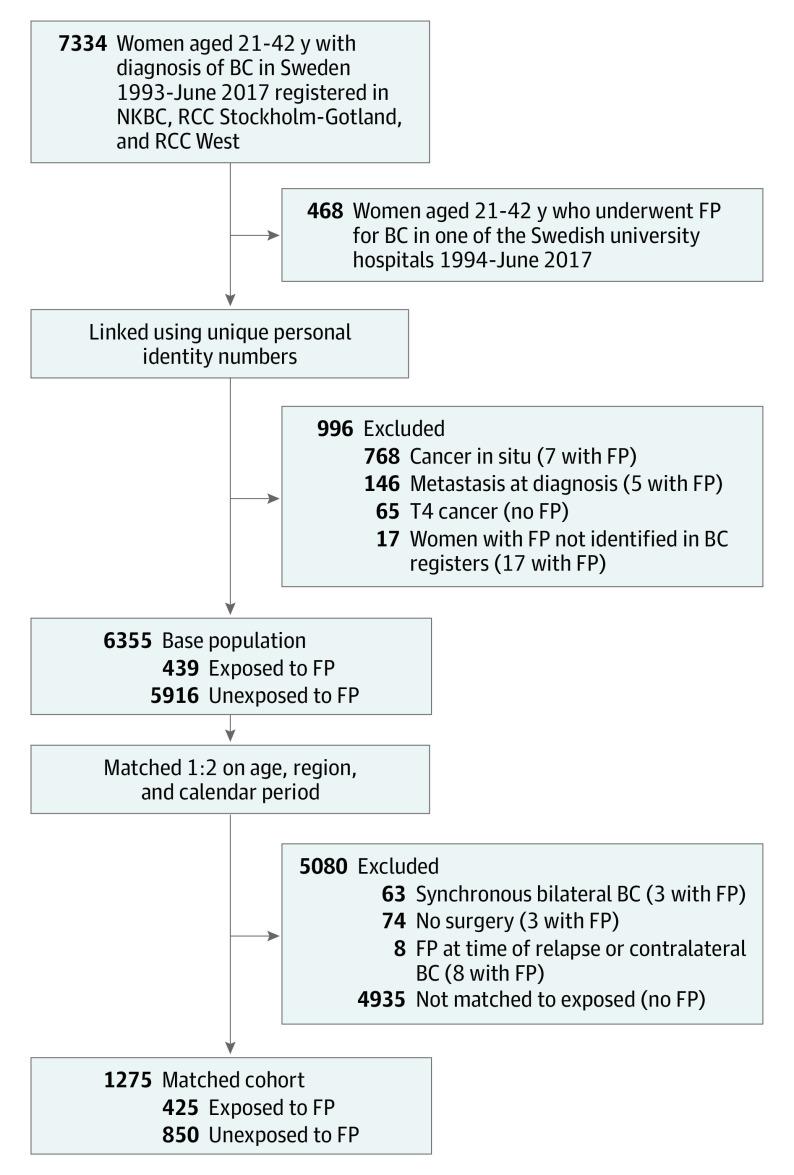
Study Diagram BC indicates breast cancer; FP, fertility preservation; NKBC, National Quality Registry for Breast Cancer; RCC, regional cancer center.

Women with cancer in situ, distant metastasis, T4 cancers, and synchronic bilateral BC, and those without BC surgery were excluded, as well as 14 women with FP who could not be identified in any BC register because their indication for FP was not BC but *BRCA* mutations, leaving 1275 women eligible for the study (Figure 1). The cohort was thereafter linked to several Swedish population registers (eTable 1 in the Supplement) using the unique personal identity number assigned to all Swedish residents.^31^ The following data were retrieved: (1) date of BC diagnosis, (2) age at diagnosis, (3) tumor characteristics, (4) BC treatment details, (5) highest attained educational level, (6) country of birth, (7) year of all live births before and after BC diagnosis, (8) date of death and migration, and (9) cause of death. For part of the cohort (women diagnosed in the Stockholm-Gotland region between 1994 and 2017 and in the West region until 2008), detailed data were available, including the date of diagnosis of ipsilateral, regional, and distant recurrence of BC. Assessment of relapse-free survival was thus possible only for this part of the cohort (later referred to as a subcohort) that included 723 women. For the rest of the cohort, the Swedish National Quality Register could not provide BC relapse-related data.

### Exposure

The main exposure was FP categorized into 2 groups: (1) cryopreservation of oocytes and/or embryos using hormonal stimulation (hormonal FP) and (2) cryopreservation of ovarian tissue without hormonal stimulation (nonhormonal FP). A combination of both methods was categorized as hormonal FP. Hormonal stimulation was further stratified by coadministration of letrozole or standard stimulation protocols.

### Outcome

The primary outcome was disease-specific mortality (ie, death due to BC) for the whole cohort. The secondary outcome was any event of death due to BC or relapse (local, regional, or systemic recurrence). Data on BC mortality were available for the whole cohort, while data on relapse were available only for the subcohort. Cancer-specific survival was defined as the time until death due to BC and relapse-free survival as the time until relapse or death due to BC.

### Statistical Analysis

Cancer-specific and relapse-free survival were estimated using the Kaplan-Meier method and presented graphically. Hazard ratios (HRs) and 95% CIs comparing BC-specific mortality and relapse in women exposed to FP with and without hormonal stimulation with those of unexposed women were estimated using Cox models stratified by the matching variables age, calendar period, and region, categorized according to Table 1. The underlying timescale was time since diagnosis, and person-years at risk were accrued from the date of BC diagnosis until the date of the event of interest (ie, BC-specific death or relapse) or censored at date of death for cause other than BC, contralateral BC, second primary nonbreast invasive cancer, emigration, or end of follow-up (December 31, 2017), whichever occurred first. Adjusted models included parity at diagnosis (0, 1, ≥2), country of birth (Nordic, non-Nordic), education level (compulsory school, secondary school, higher education), tumor size (T0, T1, T2, T3), number of lymph node metastases (0, 1-3, >3), and estrogen receptor status (positive, negative). Adjusting for chemotherapy was not possible owing to few events among women who did not receive chemotherapy. Sensitivity analyses were performed by estimating HRs for BC-related mortality and relapse separately for women exposed to hormonal FP with and without letrozole, and for shorter- and longer-term follow-up by splitting the risk time into 2 groups (<5 and ≥5 years from diagnosis). Interactions between hormonal FP with and without letrozole and each outcome, and between follow-up time and each outcome, were tested using likelihood ratio tests. The proportional hazards assumption was formally evaluated using the Schoenfeld residuals from the adjusted models. All statistical tests were 2-sided with a significance level of *P* = .05. The analyses were performed using Stata, version 17 (StataCorp).

**Table 1.** Characteristics of Women With Breast Cancer in the Matched Cohort

Characteristic	No. (%)			*P* value^a^
	Hormonal FP (n = 367)	Nonhormonal FP (n = 58)	Unexposed to FP (n = 850)	
Age at diagnosis, y				
21-24	11 (3.0)	2 (3.4)	11 (1.3)	<.001
25-29	91 (24.8)	11 (19.0)	117 (13.8)	
30-34	148 (40.3)	31 (53.4)	379 (44.6)	
35-39	112 (30.5)	14 (24.1)	333 (39.2)	
40-42	5 (1.4)	0	10 (1.2)	
Year of diagnosis				
1994-2001	11 (3.0)	7 (12.1)	36 (4.2)	<.001
2002-2004	23 (6.3)	8 (13.8)	62 (7.3)	
2005-2007	15 (4.1)	8 (13.8)	46 (5.4)	
2008-2010	50 (13.6)	9 (15.5)	118 (13.9)	
2011-2013	103 (28.1)	22 (37.9)	250 (29.4)	
2014-2017	165 (45.0)	4 (6.9)	338 (39.8)	
Geographical region				
Stockholm-Gotland	185 (50.4)	41 (70.7)	452 (53.2)	.03
West	62 (16.9)	2 (3.4)	128 (15.1)	
Other	120 (32.7)	15 (25.9)	270 (31.8)	
Educational level				
Compulsory school	23 (6.3)	4 (6.9)	84 (9.9)	.33
Secondary school	135 (36.8)	16 (27.6)	290 (34.1)	
Higher education	206 (56.1)	38 (65.5)	470 (55.3)	
Missing	3 (0.8)	0	6 (0.7)	
Country of birth				
Nordic	293 (79.8)	52 (89.7)	628 (73.9)	.004
Non-Nordic	74 (20.2)	6 (10.3)	222 (26.1)	
Parity at diagnosis				
Nulliparous	264 (71.9)	43 (74.1)	191 (22.5)	<.001
1 child	86 (23.4)	13 (22.4)	191 (22.5)	
≥2 children	17 (4.6)	2 (3.4)	468 (55.1)	
Tumor size				
T0	14 (3.8)	2 (3.4)	35 (4.1)	.05
T1	164 (44.7)	19 (32.8)	351 (41.3)	
T2	160 (43.6)	30 (51.7)	341 (40.1)	
T3	28 (7.6)	6 (10.3)	119 (14.0)	
TX	1 (0.3)	1 (1.7)	4 (0.5)	
Lymph nodes with metastasis				
0	235 (64.0)	36 (62.1)	478 (56.2)	.04
1-3	107 (29.2)	13 (22.4)	271 (31.9)	
>3	25 (6.8)	9 (15.5)	99 (11.6)	
Missing	0	0	2 (0.2)	
Tumor grade				
1	26 (7.1)	0 (0.0)	51 (6.0)	.02
2	99 (27.0)	9 (15.5)	177 (20.8)	
3	152 (41.4)	28 (48.3)	359 (42.2)	
Missing	90 (24.5)	21 (36.2)	263 (30.9)	
Estrogen receptor status				
Positive	256 (69.8)	33 (56.9)	515 (60.6)	.03
Negative	105 (28.6)	23 (39.7)	313 (36.8)	
Missing	6 (1.6)	2 (3.4)	22 (2.6)	
Progesterone receptor status				
Positive	225 (61.3)	24 (41.4)	431 (50.7)	.004
Negative	135 (36.8)	32 (55.2)	397 (46.7)	
Missing	7 (1.9)	2 (3.4)	22 (2.6)	
*ERBB2* status				
Amplified	97 (26.4)	11 (19.0)	176 (20.7)	.04
Nonamplified	176 (48.0)	23 (39.7)	429 (50.5)	
Missing	94 (25.6)	24 (41.4)	245 (28.8)	
Neoadjuvant chemotherapy				
Yes	86 (23.4)	19 (32.8)	245 (28.8)	.16
No	278 (75.7)	38 (65.5)	590 (69.4)	
Missing	3 (0.8)	1 (1.7)	15 (1.8)	
Adjuvant chemotherapy				
Yes	277 (75.5)	44 (75.9)	562 (66.1)	.005
No	90 (24.5)	13 (22.4)	281 (33.1)	
Missing	0	1 (1.7)	7 (0.8)	
Radiotherapy				
Yes	275 (74.9)	42 (72.4)	650 (76.5)	.004
No	83 (22.6)	11 (19.0)	141 (16.6)	
Missing	9 (2.5)	5 (8.6)	59 (6.9)	

Abbreviation: FP, fertility preservation.

^a^ *P* values estimated using the Kruskal-Wallis test for median age and the Pearson χ^2^ test for categorical variables.

## Results

### Study Population

The final study population included 1275 women aged 21 to 42 years at the time of BC diagnosis, whereof 425 women received FP treatments (cryopreservation of ovarian tissue in 58 women, COS for cryopreservation of oocytes or/and embryos in 362 women, and a combination of these methods in 5 women; Figure 1). Baseline characteristics of the cohort are summarized in Table 1. Women who did not undergo FP had higher parity (191 of 850 women [22.5%] were nulliparous compared with 264 of 367 women [71.9%] in the hormonal FP group and 43 of 58 women [74.1%] in nonhormonal FP group), were slightly older (median age of 34 years compared with 33 years in the hormonal FP group and 32 years in the nonhormonal FP group), and were more likely to be born outside of Scandinavia (222 of 850 women [26.1%] compared with 74 of 367 women [20.2%] in the hormonal FP group and 6 of 58 women [10.3%] in the nonhormonal FP group), while the educational level was similar among the groups. The majority of women who underwent nonhormonal FP were from the region of Stockholm (41 of 58 women [70.7%]). There were certain differences in biological characteristics and treatment modalities among the groups, but no pattern of generally more aggressive disease could be seen for any group compared with the other 2 (Table 1).

The risk of relapse was investigated in the subcohort of 723 women, of whom 198 were exposed to hormonal FP, 43 to nonhormonal FP, and 482 were unexposed. Baseline characteristics of the subcohort are presented in eTable 2 in the Supplement.

### Disease-Specific Mortality

Death due to BC occurred in 17 women who underwent hormonal FP, 7 women who underwent nonhormonal FP, and 80 women who were unexposed to FP (Table 2). The median follow-up was 4.0 years among women who underwent hormonal FP and 3.8 years in their matched controls, and 6.7 years in women who underwent nonhormonal FP and 6.7 years among their matched controls.

**Table 2.** Risk of Cancer-Specific Mortality and Relapse in Women Who Underwent Fertility Preservation (FP) With and Without Hormonal Stimulation Compared With Women Who Did Not

Variable	No. of events	Person-years	Hazard ratio (95% CI)	
			Univariate^a^	Adjusted^b^
Breast cancer mortality				
Unexposed to FP	80	4262	1 [Reference]	1 [Reference]
Exposed to FP				
Without hormonal stimulation	7	475	0.70 (0.32-1.54)	0.51 (0.20-1.29)
With hormonal stimulation	17	1841	0.51 (0.30-0.89)	0.59 (0.32-1.09)
Relapse or breast cancer mortality^c^				
Unexposed to FP	97	2847	1 [Reference]	1 [Reference]
Exposed to FP				
Without hormonal stimulation	9	367	0.78 (0.39-1.57)	0.75 (0.35-1.62)
With hormonal stimulation	26	1211	0.70 (0.45-1.09)	0.81 (0.49-1.37)

^a^ Stratified by age, calendar period, and region, and adjusted for time since diagnosis (as timescale).

^b^ Stratified by age, calendar period, and region, and adjusted for time since diagnosis (as timescale), country of birth, education, parity at diagnosis, tumor size, number of lymph node metastases, and estrogen receptor status.

^c^ In the subcohort of patients with complete relapse information (n = 723).

In univariate analyses (Table 2), disease-specific mortality was lower among women who underwent hormonal FP (HR, 0.51; 95% CI, 0.30-0.89) compared with women who had not. However, after adjustment for potential confounders this difference was not statistically significant (adjusted HR [aHR], 0.59; 95% CI, 0.32-1.09). In women receiving nonhormonal FP, the disease-specific mortality rate was 0.70 (95% CI, 0.32-1.54) in univariate analysis and 0.51 (95% CI, 0.20-1.29) after adjustment when compared with women who were unexposed to FP.

### Disease-Specific Mortality and Relapse

In the subcohort of 723 women with detailed information on relapse, BC-related death or relapse occurred in 97 women unexposed to FP, 9 women who underwent nonhormonal FP, and 26 women who underwent hormonal FP. The median follow-up was 5.3 years in women who underwent hormonal FP and 4.9 years in their matched controls, and 7.0 years in women who underwent nonhormonal FP and 6.9 years in their controls. There was no statistically significant difference in the rate of relapse or death among women who underwent hormonal FP vs women who underwent nonhormonal FP in either univariate analysis (HR, 0.70; 95% CI, 0.45-1.09 vs HR, 0.78; 95% CI, 0.39-1.57) or after adjustment for potential confounders (aHR, 0.81; 95% CI, 0.49-1.37 vs aHR, 0.75; 95% CI, 0.35-1.62).

### Disease-Specific and Relapse-Free Survival

**Figure 2.**
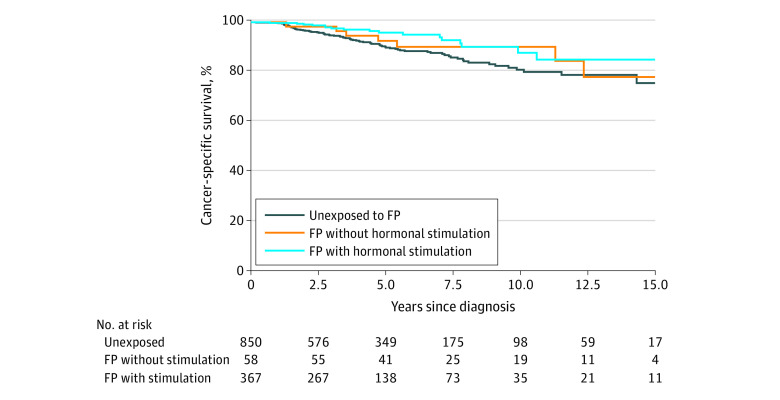
Cancer-Specific Survival in the Cohort FP indicates fertility preservation.

**Figure 3.**
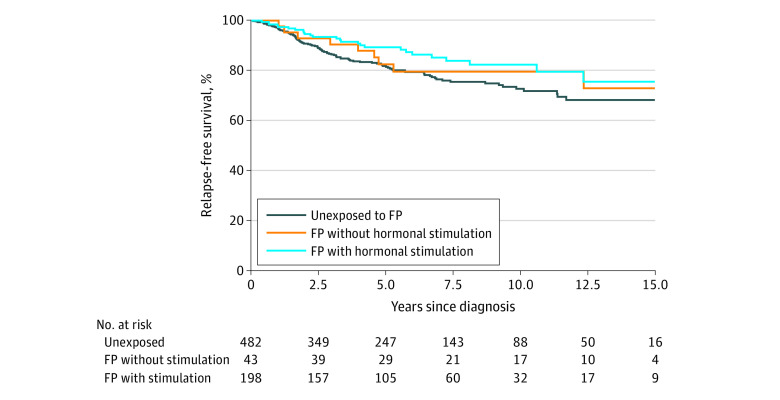
Relapse-Free Survival in the Subcohort FP indicates fertility preservation.

The 5-year BC-specific survival rate was 96% in the group that underwent hormonal FP, 93% in the group that underwent nonhormonal FP, and 90% in the unexposed group. Corresponding estimates after 10 years were 88%, 90%, and 81%, respectively (Figure 2). The 5-year relapse-free survival rate was 89% in women who underwent hormonal FP, 83% in women who underwent nonhormonal FP, and 82% in women who did not receive FP. After 10 years, the corresponding estimates were 82%, 80%, and 73%, respectively (Figure 3).

### Sensitivity Analyses

Among 367 women exposed to FP with hormonal stimulation, 207 women received COS with coadministration of letrozole, 141 women received COS without letrozole, and for 19 women the stimulation protocol was unknown. When comparing the groups who had undergone hormonal stimulation for FP with and without letrozole, there was no statistically significant difference in BC-related mortality (aHR, 0.45; 95% CI, 0.13-1.56 vs aHR, 0.59; 95% CI, 0.29-1.20; *P* = .69) nor BC-related mortality and relapse (aHR, 0.98; 95% CI, 0.41-2.33 vs aHR, 0.72; 95% CI, 0.38-1.34; *P* = .54) (eTable 3 in the Supplement).

When estimating HRs separately by length of follow-up time, there were no statistically significant differences in either BC-related mortality or BC mortality and relapse within vs after 5 years from diagnosis (eTable 4 in the Supplement). The aHR of BC-related mortality for women receiving FP with hormonal stimulation was 0.53 (95% CI, 0.25-1.11) within 5 years from diagnosis and 0.74 (95% CI, 0.28-1.97) after 5 years compared with unexposed women (*P* = .69). The corresponding estimates for relapse or BC-related mortality were 0.75 (95% CI, 0.42-1.34) in short-term follow-up and 1.07 (95% CI, 0.43-2.67) in longer-term follow-up (*P* = .78).

## Discussion

We found similar estimates of disease-specific and relapse-free survival in women with diagnosis of BC at a young age who underwent hormonal FP, nonhormonal FP, or no FP. The results are consistent with previous findings on disease-free and overall survival after FP indicated by BC.^21,23,28,32,33^ Based on data from a nationwide prospective cohort—to our knowledge, the largest one reported to date—these results add substantially to the evidence on safety of FP procedures in women with BC.

Fertility preservation has become recognized as an important issue to discuss with patients diagnosed with cancer at reproductive age. For women, cryopreservation of oocytes and embryos is recommended as the first FP option when there is enough time to perform COS.^16^ So far, there is no evidence of a detrimental association of FP with BC prognosis, irrespective of COS protocols used, although studies have been observational and include heterogeneous study populations.^23,25,26,28,33^ Comparing the outcomes of 120 women who underwent COS with addition of letrozole and 217 women who declined FP at the time of BC diagnosis, Kim et al^28^ reported no statistically significant difference in relapse-free survival after a mean follow-up of 5.0 vs 6.9 years. Similarly, in a cohort of 157 women who received FP with a standard COS protocol and 175 women who did not, no statistically significant difference in BC recurrence or mortality was found after a median follow-up of 4.0 vs 6.2 years.^33^ In a matched cohort study including 566 young women with BC, Rodriguez-Wallberg et al^23^ reported that hormonal stimulation with or without letrozole was not associated with a higher risk of BC recurrence during a mean follow-up time of 6.6 years. In the present study, survival in women who had undergone hormonal stimulation for FP either with or without letrozole was similar to that of unexposed women. In the setting of counseling BC patients on FP options, these results may offer additional support to opt for FP, irrespective of the COS protocol used in a certain clinic.

In women with sufficient ovarian reserve, cryopreservation of ovarian tissue can be considered when chemotherapy needs to be started promptly.^34^ While retransplantation of ovarian tissue may involve a potential risk of reintroducing metastatic cancer,^35^ extraction of ovarian tissue, per se, is unlikely to affect risk of relapse or death in women with BC because it is an FP method that does not require any hormonal stimulation. We found no statistically significant difference in long-term outcomes between women who underwent nonhormonal FP and women who did not, which supports the previous statement. However, the confidence intervals were wide owing to the small numbers in this subgroup. Analysis of long-term outcomes for women who had undergone retransplantation of ovarian tissue could not be performed owing to sparse data.

In univariate analysis, the risk of BC-related mortality was slightly lower in women who underwent hormonal FP compared with unexposed women. This difference became not statistically significant after adjustment for potential confounders, indicating possible selection bias that could have occurred in the process of referral to FP clinics or at the time of making a decision about FP. As previously suggested, a “healthy FP effect” may explain improved long-term outcomes in women with a history of FP because those who feel healthier and appreciate their prognosis to be good may be more prone to opt for FP at the time of BC diagnosis.^29^

### Strengths and Limitations

Strengths of this study include the large size of the nationwide cohort, with a relevant group of matched comparators unexposed to FP at the time of BC diagnosis sampled from population-based quality registers, as well as the use of prospectively collected data from the registers (ie, with all of the possible precautions taken to avoid selection bias). Detailed data on disease and treatment characteristics obtained from the national and regional quality registers for BC are of particular importance in this context. In Sweden, reporting the data to the quality registers is obligatory and does not require informed consent, which results in an almost complete coverage. The possibility to adjust for proxies of socioeconomic status such as educational level and the country of birth is an additional strength. Equal access to anticancer treatments and FP indicated by medical reasons is provided in Sweden to all citizens within the public tax-funded system,^36,37^ which further reduces the risk of selection bias.

However, this study is not without limitations, the main one being relatively short follow-up, though to our knowledge, one of the longest reported so far. The first peak of BC recurrence occurs at 18 months after surgery, while the second comes around 60 months.^38^ Median follow-up for relapse-free survival was 4.0 years for women who underwent hormonal FP, which covers the first peak of recurrence and comes close to covering the second peak as well. As recurrence in a tapering-off pattern extends up to 15 years,^38^ longer follow-up is desirable for more definitive statements on long-term safety of FP in this population, in particular for women with estrogen receptor–positive BC.^39^ Nevertheless, the specific analyses restricted to women with follow-up longer than 5 years in this study also provide reassuring findings. The specific data on relapse-free survival were only available for the subcohort, which showed skewed follow-up times owing to the inclusion of women with BC diagnoses prior to 2008 in the West region, earlier than at the rest of Sweden. *ERBB2 *(formerly *HER2*) screening and targeted treatment with trastuzumab were introduced in Sweden in 2005, which partially explains the missing data on *ERBB2* status for more than 25% of the cohort. However, because *ERBB2 *status was often not known at the time of FP counseling, it was unlikely to influence whether FP was performed.

## Conclusions

In this large nationwide cohort study of women with BC at reproductive age, disease-specific and relapse-free survival were similar for women who underwent hormonal FP, women who underwent nonhormonal FP, and women who were unexposed to FP. Results of this study provide much needed additional evidence on the safety of FP procedures in women with BC and may influence current health care practice to the benefit of young women with BC who wish to preserve their fertility. Women diagnosed with BC during their reproductive years should be referred, when interested, for fertility counseling and provided with the available information on safety of the procedures that are offered. Future research evaluating long-term safety of FP in young women with BC should ideally include even longer follow-up.

## Notes

Supplement. **eTable 1.** Swedish population registers used to obtain information on cohort **eTable 2.** Characteristics of women with breast cancer in the part of the cohort used for analysis of relapse **eTable 3.** Risk of cancer-specific mortality and relapse in patients exposed to FP without stimulation, with letrozole stimulation or other hormonal stimulation, compared to women without FP **eTable 4. **Risk of cancer-specific mortality and relapse in patients treated with FP with and without hormonal stimulation, compared to women without FP, by length of follow-up
