# Supplementary material for: Relapse Rates and Disease-Specific Mortality Following Procedures for Fertility Preservation at Time of Breast Cancer Diagnosis
Source: JAMA Oncol. 2022 Aug 25;8(10):1438–46. doi: 10.1001/jamaoncol.2022.3677 (PMC9412846; doi:10.1001/jamaoncol.2022.3677)
Supplement: Supplement. — eTable 1. Swedish population registers used to obtain information on cohort eTable 2. Characteristics of women with breast cancer in the part of the cohort used for analysis of relapse eTable 3. Risk of cancer-specific mortality and relapse in patients exposed to FP without stimulation, with letrozole stimulation or other hormonal stimulation, compared to women without FP eTable 4. Risk of cancer-specific mortality and relapse in patients treated with FP with and without hormonal stimulation, compared to women without FP, by length of follow-up [file jamaoncol-e223677-s001.pdf]

Supplemental Online Content

Marklund A, Lekberg T, Hedayati E, et al. Relapse rates and disease-specific mortality following procedures for fertility preservation at time of breast cancer diagnosis. *JAMA Oncol*. Published online August 25, 2022.
doi:10.1001/jamaoncol.2022.3677

eTable 1. Swedish population registers used to obtain information on cohort
eTable 2. Characteristics of women with breast cancer in the part of the cohort used for analysis of relapse
eTable 3. Risk of cancer-specific mortality and relapse in patients exposed to FP without stimulation, with letrozole stimulation or other hormonal stimulation, compared to women without FP
eTable 4. Risk of cancer-specific mortality and relapse in patients treated with FP with and without hormonal stimulation, compared to women without FP, by length of follow-up

This supplemental material has been provided by the authors to give readers additional information about their work.

© 2022 Marklund A et al. *JAMA Oncol.*

eTable 1. Swedish population registers used to obtain information on cohort

Register	Description	Years	Information retrieved
Swedish National Cancer Register	Started in 1958 and includes data on histological type, site, date of diagnosis, eventual date and cause of death, with a coverage rate of 96%.[1]	1994-2017	Date of BC diagnosis, age at diagnosis, tumor characteristics, treatment details for BC, date of relapse
Swedish National Quality Register for BC	A population-based register, with information on tumor characteristics, treatment, and relapse occurrence in patients diagnosed with invasive BC since 2008, with a coverage rate of 99%.[2]	2008-2017	
Regional quality registers for BC, for regions West and Stockholm-Gotland	Prior to 2008, data on tumor characteristics, treatment details and relapse occurrence for all cases of invasive BC were reported to the six regional quality registers.	1994-2007	
The Total Population Register	It contains data on life events including birth, death, marital status and migration; maintained by the government agency Statistics Sweden. Updates are transmitted daily from the Tax Agency to the register.[3]	1994-2018	Date of death, immigrations and emigrations
The Cause of Death Register	The Swedish cause of death register is a high quality virtually complete register of all deaths in Sweden since 1952.[4]	1994-2017	Underlying cause of death, recorded according to the current version of the ICD classification
The Swedish Multi-Generation Register	It contains connections between index persons (registered in Sweden at some time since 1961 and born in 1932 or later) and their biological parents.[5]	1994-2018	Year of live births
Longitudinal integrated database for health insurance and labor market studies (LISA)	It is maintained by the government agency Statistics Sweden and holds annual registers since 1990, integrating existing data from the labor market, educational and social sector.[6]	1994-2017	Educational level and country of birth
Abbreviations: BC, breast cancer.			

References:
1. Socialstyrelsen. Swedish National Cancer Register. Available from: https://www.socialstyrelsen.se/en/statistics-and-data/registers/register-information/swedish-cancer-register/. [Accessed 1st June 2020]
2. Lofgren L, Eloranta S, Krawiec K, Asterkvist A, Lonnqvist C, Sandelin K, et al. Validation of data quality in the Swedish National Register for Breast Cancer. BMC Public Health. 2019;19(1):495.
3. Ludvigsson JF, Almqvist C, Bonamy AK, Ljung R, Michaelsson K, Neovius M, et al. Registers of the Swedish total population and their use in medical research. Eur J Epidemiol. 2016;31(2):125-36.
4. Brooke HL, Talback M, Hornblad J, Johansson LA, Ludvigsson JF, Druid H, et al. The Swedish cause of death register. Eur J Epidemiol. 2017;32(9):765-73.
5.Ekbom A. The Swedish Multi-generation Register. Methods Mol Biol. 2011;675:215-20.
6. Ludvigsson JF, Svedberg P, Olen O, Bruze G, Neovius M. The longitudinal integrated database for health insurance and labour market studies (LISA) and its use in medical research. Eur J Epidemiol. 2019;34(4):423-37.

eTable 2. Characteristics of women with breast cancer in the part of the cohort used for analysis of relapse

	Hormonal FP (n= 198)	Non-hormonal FP (n=43)	Unexposed to FP (n=482)	p-value χ^2-test
**Age at diagnosis**				0.016
21-24 y	4 (2.0%)	1 (2.3%)	9 (1.9%)	
25-29 y	50 (25.3%)	9 (20.9%)	66 (13.7%)	
30-34 y	79 (39.9%)	21 (48.8%)	196 (40.7%)	
35-39 y	61 (30.8%)	12 (27.9%)	203 (42.1%)	
40-42 y	4 (2.0%)	0 (0.0%)	8 (1.7%)	
**Year of diagnosis**				0.07
1994-2001	11 (5.6%)	7 (16.3%)	36 (7.5%)	
2002-2004	23 (11.6%)	8 (18.6%)	62 (12.9%)	
2005-2007	15 (7.6%)	8 (18.6%)	46 (9.5%)	
2008-2010	41 (20.7%)	7 (16.3%)	96 (19.9%)	
2011-2013	52 (26.3%)	9 (20.9%)	122 (25.3%)	
2014-2017	56 (28.3%)	4 (9.3%)	120 (24.9%)	
**Geographical region**				0.90
Stockholm Gotland	185 (93.4%)	41 (95.3%)	452 (93.8%)	
West region	13 (6.6%)	2 (4.7%)	30 (6.2%)	
**Educational level**				0.09
Compulsory school	12 (6.1%)	0 (0.0%)	55 (11.4%)	
Secondary shool	64 (32.3%)	14 (32.6%)	161 (33.4%)	
Higher education	120 (60.6%)	29 (67.4%)	263 (54.6%)	
Missing	2 (1.0%)	0 (0.0%)	3 (0.6%)	
**Country of birth**				0.01
Nordic	159 (80.3%)	38 (88.4%)	348 (72.2%)	
Non-Nordic	39 (19.7%)	5 (11.6%)	134 (27.8%)	
**Parity at diagnosis**				<0.001
Nulliparous	141 (71.2%)	33 (76.7%)	109 (22.6%)	
1 child	48 (24.2%)	10 (23.3%)	104 (21.6%)	
≥2 children	9 (4.5%)	0 (0.0%)	269 (55.8%)	
**Tumour size**				0.15
T0	6 (3.0%)	1 (2.3%)	21 (4.4%)	
T1	92 (46.5%)	15 (34.9%)	199 (41.3%)	
T2	85 (42.9%)	20 (46.5%)	190 (39.4%)	
T3	15 (7.6%)	6 (14.0%)	69 (14.3%)	
TX (cannot be assessed)	0 (0.0%)	1 (2.3%)	3 (0.6%)	
**Lymph nodes with metastasis**				0.24
0	128 (64.6%)	26 (60.5%)	271 (56.2%)	
1-3	58 (29.3%)	11 (25.6%)	153 (31.7%)	
>3	12 (6.1%)	6 (14.0%)	57 (11.8%)	
Missing	0 (0.0%)	0 (0.0%)	1 (0.2%)	

© 2022 Marklund A et al. *JAMA Oncol.*

**Tumor Grade**				**0.009**
1	17 (8.6%)	0 (0.0%)	22 (4.6%)	
2	51 (25.8%)	4 (9.3%)	98 (20.3%)	
3	73 (36.9%)	20 (46.5%)	178 (36.9%)	
Missing	57 (28.8%)	19 (44.2%)	184 (38.2%)	
**ER-status**				0.34
Positive	134 (67.7%)	22 (51.2%)	312 (64.7%)	
Negative	59 (29.8%)	20 (46.5%)	158 (32.8%)	
Missing	5 (2.5%)	1 (2.3%)	12 (2.5%)	
**PR-status**				0.04
Positive	118 (59.6%)	15 (34.9%)	255 (52.9%)	
Negative	74 (37.4%)	27 (62.8%)	215 (44.6%)	
Missing	6 (3.0%)	1 (2.3%)	12 (2.5%)	
**HER-2**				0.06
Amplified	43 (21.7%)	7 (16.3%)	89 (18.5%)	
Non-amplified	88 (44.4%)	13 (30.2%)	235 (48.8%)	
Missing	67 (33.8%)	23 (53.5%)	158 (32.8%)	
**Neoadjuvant chemotherapy**				0.10
Yes	49 (24.7%)	17 (39.5%)	158 (32.8%)	
No	146 (73.7%)	25 (58.1%)	309 (64.1%)	
Missing	3 (1.5%)	1 (2.3%)	15 (3.1%)	
**Adjuvant chemotherapy**				0.01
Yes	147 (74.2%)	30 (69.8%)	298 (61.8%)	
No	51 (25.8%)	12 (27.9%)	180 (37.3%)	
Missing	0 (0.0%)	1 (2.3%)	4 (0.8%)	
**Radiotherapy**				0.11
Yes	157 (79.3%)	33 (76.7%)	384 (79.7%)	
No	33 (16.7%)	6 (14.0%)	55 (11.4%)	
Missing	8 (4.0%)	4 (9.3%)	43 (8.9%)	

p-values estimated using the Kruskal-Wallis test for median age and the Pearson chi-square test for categorical variables.

© 2022 Marklund A et al. *JAMA Oncol.*

eTable 3. Risk of cancer-specific mortality and relapse in patients exposed to FP without stimulation, with letrozole stimulation or other hormonal stimulation, compared to women without FP

	N of events	Person years	Univariate [a] HR (95% CI)	Adjusted [b] HR (95% CI)
**Breast cancer mortality [d]**				
Unexposed to FP	80	4262	1.00 (ref.)	1.00 (ref.)
Non-hormonal FP	7	475	0.70 (0.32-1.54)	0.51 (0.20-1.29)
Hormonal FP with letrozole	3	619	0.39 (0.12-1.29)	0.45 (0.13-1.56)
Hormonal FP without letrozole	11	1051	0.53 (0.27-1.01)	0.59 (0.29-1.20)
**Relapse or breast cancer mortality [e]**				
Unexposed to FP	97	2847	1.00 (ref.)	1.00 (ref.)
Non-hormonal FP	9	367	0.77 (0.38-1.54)	0.74 (0.34-1.61)
Hormonal FP with letrozole	8	293	0.86 (0.38-1.91)	0.98 (0.41-2.33)
Hormonal FP without letrozole	15	798	0.62 (0.35-1.08)	0.72 (0.38-1.34)

Abbreviations: CI, confidence interval; FP, fertility preservation; HR, hazard ratio

[a] Stratified by age, calendar period and region, adjusted for time since diagnosis (as timescale)

[b] Stratified by age, calendar period and region, adjusted for time since diagnosis (as timescale), country of birth, education, parity at diagnosis, tumor size, lymph node metastases, estrogen receptor status

[d] In the subcohort of patients with information on stimulation protocol, n=1256. Likelihood-ratio test of interaction with letrozole, adjusted p-value 0.69

[e] In the subcohort of patients with information on stimulation protocol and relapse, n=711. Likelihood-ratio test of interaction with letrozole, adjusted p-value 0.54

© 2022 Marklund A et al. *JAMA Oncol.*

eTable 4. Risk of cancer-specific mortality and relapse in patients treated with FP with and without hormonal stimulation, compared to women without FP, by length of follow-up

	Follow-up less than 5 years				Follow-up 5 years or more			
	N of events	Person years	Univariate [a] HR (95% CI)	Adjusted [b] HR (95% CI)	N of events	Person years	Univariate [a] HR (95% CI)	Adjusted [b] HR (95% CI)
**Breast cancer mortality [c]**								
Unexposed to FP	57	2966	1.00 (ref.)	1.00 (ref.)	23	1296	1.00 (ref.)	1.00 (ref.)
Non-hormonal FP	4	268	0.68 (0.24-1.93)	0.46 (0.13-1.65)	3	206	0.74 (0.22-2.52)	0.57 (0.15-2.16)
Hormonal FP	10	1320	0.42 (0.21-0.83)	0.53 (0.25-1.11)	7	521	0.79 (0.31-1.98)	0.74 (0.28-1.97)
**Relapse or breast cancer mortality [d]**								
Unexposed to FP	73	1786	1.00 (ref.)	1.00 (ref.)	24	1061	1.00 (ref.)	1.00 (ref.)
Non-hormonal FP	7	191	0.82 (0.37-1.81)	0.76 (0.32-1.81)	2	176	0.65 (0.15-2.87)	0.70 (0.14-3.37)
Hormonal FP	18	781	0.63 (0.37-1.07)	0.75 (0.42-1.34)	8	430	0.91 (0.40-2.11)	1.07 (0.43-2.67)

Abbreviations: CI, confidence interval; FP, fertility preservation; HR, hazard ratio

[a] Stratified by age, calendar period and region, adjusted for time since diagnosis (as timescale)

[b] Stratified by age, calendar period and region, adjusted for time since diagnosis (as timescale), country of birth, education, parity at diagnosis, tumor size, lymph node metastases, estrogen receptor status

[c] Likelihood-ratio test of interaction with follow-up time, adjusted p-value 0.69

[d] In the subcohort of patients with complete relapse information, n = 723. Likelihood-ratio test of interaction with follow-up time, adjusted p-value 0.78

© 2022 Marklund A et al. *JAMA Oncol.*
